# Withdrawal Syndrome Following Opioid Rotation: Tramadol and Its Unique Pharmacology

**DOI:** 10.7759/cureus.102584

**Published:** 2026-01-29

**Authors:** Matthew A Murphy

**Affiliations:** 1 Department of Supportive Care Medicine, Moffitt Cancer Center, Tampa, USA

**Keywords:** cancer pain, chronic myeloid leukemia, oncology, opioid conversion, opioid rotation, opioid withdrawal, palliative care, tramadol

## Abstract

A 33-year-old woman with chronic myeloid leukemia and a history of generalized anxiety disorder and major depressive disorder presented to the hospital with severe anxiety. Two days earlier, she reported escalating cancer-related pain despite taking tramadol 100 mg orally three times per day. Following opioid rotation to oxycodone 5 mg orally every six hours as needed, her pain improved, but she developed anxiety and restlessness consistent with opioid withdrawal, prompting hospital evaluation. Her symptoms resolved following opioid dose escalation to oxycodone 10 mg orally every four hours as needed. This case highlights the potential for withdrawal during opioid rotation and underscores tramadol’s unique pharmacology, which may differentiate its withdrawal syndrome from that of other opioid analgesics.

## Introduction

Pain occurs in nearly half of individuals with cancer and can significantly limit function and negatively impact quality of life [1]. Opioids are commonly prescribed for the management of moderate-to-severe cancer pain, with the dual aim of achieving adequate analgesia and meeting patient goals [2]. Whenever possible, opioids are prescribed as one part of a multimodal analgesic plan, leveraging different mechanisms of action across classes of analgesic medications. Safe and effective opioid use within a multimodal analgesic framework requires an understanding of opioid pharmacology and the application of clinical judgment tailored to the individual patient.

Opioids primarily exert their analgesic effect through agonism of mu-opioid receptors within the central and peripheral nervous systems, which inhibits nociception and modulates pain perception [3]. Although opioid analgesics share this fundamental mechanism of action, individual opioids differ in receptor affinity, metabolic pathways, and pharmacokinetic profiles. Additionally, opioid metabolism can vary substantially between individuals based on genotype, particularly due to polymorphisms in cytochrome P450 enzymes such as CYP2D6, resulting in clinically meaningful differences in analgesic efficacy and risk of adverse effects [4]. As such, an understanding of opioid pharmacology is essential for clinicians who manage cancer-related pain.

Opioid rotation refers to the clinical practice of switching from one opioid to another or changing the route of administration. Rotation between opioid analgesics may be advised in several situations, for example, a patient may have inadequate pain control or develop intolerable adverse effects with their current opioid regimen. Patients may lose the ability to swallow, requiring rotation to an opioid that can be administered via a gastrostomy tube or via the transdermal route. Practical considerations, including cost and the availability of specific opioids in local pharmacies, can likewise prompt the need for opioid rotation [5]. When performing an opioid rotation, it is critical that clinicians understand the concept of equianalgesia, which refers to achieving an equipotent analgesic effect when switching between different opioids or routes of administration. If the dose of the new opioid is too high, it can result in adverse effects such as sedation or respiratory depression, whereas if the dose is too low, it may result in poor analgesia and precipitate symptoms of opioid withdrawal.

Tramadol is a synthetic opioid with a dual mechanism of action. Like other opioid analgesics, tramadol acts as a mu-opioid receptor agonist. However, unlike most opioids, tramadol also inhibits monoaminergic reuptake [6], which may contribute to a more prolonged and atypical withdrawal syndrome when it is suddenly discontinued. Understanding these nuances is critical for clinicians who perform opioid rotation from tramadol to other opioid analgesics in patients with cancer. This case emphasizes the essential role of equianalgesia in limiting the risk of withdrawal during opioid rotation and highlights the unique monoaminergic-mediated effects associated with abrupt tramadol discontinuation.

## Case presentation

A 33-year-old woman with chronic myeloid leukemia and a history of generalized anxiety disorder and major depressive disorder presented to the hospital with severe anxiety that began the day prior to presentation. Two days earlier, she reported escalating cancer-related pain despite taking tramadol 100 mg orally three times per day, which she had been taking for several months. Due to inadequate analgesia, the patient’s opioid regimen was rotated from tramadol 100 mg orally every six hours as needed to oxycodone 5 mg orally every six hours as needed. The patient reported acceptable analgesia after rotation to oxycodone but began to experience escalating anxiety and restlessness that were refractory to lorazepam 0.5 mg orally, as prescribed by her psychiatrist. The patient contacted the on-call palliative care physician and was advised to present to the hospital for evaluation due to concern for opioid withdrawal.

During the interview, the patient noted no significant changes in her overall medical condition and no recent stressors or trauma that she believed contributed to her mood change. She reported no medication changes other than the recent rotation from tramadol to oxycodone. Current medications included asciminib 40 mg orally daily, buspirone 10 mg orally twice daily, fluoxetine 20 mg orally daily, lorazepam 0.5 mg orally twice daily as needed for anxiety, and oxycodone 5 mg orally every six hours as needed for pain. The patient denied alcohol, tobacco, or illicit drug use.

On physical examination, vital signs were within normal limits (Table 1). The patient appeared anxious without psychosis or suicidal ideation; the remainder of the examination was normal. Complete blood count, basic metabolic panel, and thyroid function tests were obtained and demonstrated no clinically significant abnormalities (Table 2).

**Table 1:** Patient vital signs. mmHg: Millimeters of mercury; bpm: Beats per minute; min: Minute; °F: Fahrenheit.

Vital sign	Value	Reference range
Blood pressure	124/84 mmHg	90-120/60-80 mmHg
Heart rate	100 bpm	60-100 bpm
Respiratory rate	16/min	12-20/min
Temperature	98.1 °F	97-99 °F

**Table 2:** Laboratory studies. k/µL: Thousand cells per microliter; g/dL: Grams per deciliter; mmol/L: Millimoles per liter; mg/dL: Milligrams per deciliter; µIU/mL: Micro-international units per milliliter; pg/mL: Picograms per milliliter; ng/dL: Nanograms per deciliter; BUN: Blood urea nitrogen; TSH: Thyroid-stimulating hormone; T3: Triiodothyronine; T4: Thyroxine.

Test	Result	Reference range
WBCs	10.45 k/µL	4.0-11.0 k/µL
Hemoglobin	15.8 g/dL	12.0-15.5 g/dL (female)
Hematocrit	45.20%	36-46% (female)
Platelets	325,000/µL	150,000-450,000/µL
Sodium	138 mmol/L	135-145 mmol/L
Potassium	4.3 mmol/L	3.5-5.0 mmol/L
Chloride	107 mmol/L	96-108 mmol/L
Bicarbonate	19 mmol/L	22-28 mmol/L
BUN	10 mg/dL	7-20 mg/dL
Creatinine	0.75 mg/dL	0.6-1.3 mg/dL
Glucose	143 mg/dL	≤140 mg/dL
TSH	1.502 µIU/mL	0.4-4.0 µIU/mL
Free T3	2.63 pg/mL	2.3-4.2 pg/mL
Free T4	0.97 ng/dL	0.8-1.8 ng/dL

The patient received hydromorphone 0.5 mg IV, resulting in rapid improvement in anxiety. Based on this response, the palliative care team attributed her symptoms to opioid withdrawal rather than depressed monoaminergic activity. Her opioid regimen was adjusted to oxycodone 10 mg orally every four hours as needed for pain or restlessness. During hospitalization, she received two additional doses of oxycodone 10 mg orally and reported adequate analgesia with complete resolution of anxiety symptoms. She was discharged later that day with a scheduled follow-up in the palliative care clinic in one week. During her follow-up visit, the patient continued to endorse adequate pain control and reported no symptoms of anxiety.

## Discussion

Patients receiving chronic daily opioid therapy may develop tolerance, often described colloquially as “the body gets used to the substance.” Opioid tolerance is commonly defined as daily opioid use for at least 7 days at or above specified minimum doses (Table 3) [7]. When tolerance occurs, patients exhibit a diminished analgesic response to their current daily opioid dose despite no substantial change in their underlying condition. In these situations, one strategy to improve analgesia is to perform opioid rotation to a different opioid at a relatively equianalgesic dose. This approach leverages differences in opioid receptor binding sites and affinities, which may result in improved analgesia.

**Table 3:** Minimum daily doses of common opioid analgesics that define opioid tolerance after at least seven consecutive days of therapy. PO: By mouth; TD: Transdermal; mg: Milligrams; mcg/h: Micrograms per hour.

Opioid (route)	Minimum daily dose
Morphine (PO)	60 mg
Oxycodone (PO)	30 mg
Hydromorphone (PO)	8 mg
Hydrocodone (PO)	60 mg
Fentanyl (TD)	25 mcg/h
Oxymorphone (PO)	25 mg

Chronic daily opioid use may also lead to physical dependence, defined as a state in which abrupt dose reduction or discontinuation precipitates a withdrawal syndrome characterized by anxiety, restlessness, insomnia, diaphoresis, nausea/vomiting, diarrhea, and myalgias [8]. In the present case, withdrawal symptoms occurred following opioid rotation from tramadol to oxycodone at a comparatively reduced daily dose.

Various methods exist to guide opioid rotation [9]. One common approach consists of calculating the patient’s total daily opioid exposure, converting this dose to oral morphine equivalents (OME) using an equianalgesic table, and subsequently converting from OME to the desired opioid. An equianalgesic table is a reference that provides approximate dose equivalents between different opioid analgesics and routes of administration, which are expected to produce a similar degree of analgesia [10]. Importantly, there is no universally agreed-upon equianalgesic table, and conversion ratios derived from such tables should be viewed as approximations that do not account for individual patient variability. Accordingly, when rotating opioids in opioid-tolerant patients, clinicians also frequently reduce the calculated dose to account for incomplete cross-tolerance, recognizing that tolerance to one opioid may not fully apply to another. Performing a 1:1 conversion without dose reduction may increase the risk of adverse effects such as sedation or respiratory depression. Some experts recommend a 25-50% opioid dose reduction [11,12]; however, a recent international consensus panel could not reach agreement on this practice [13]. While dose reduction may enhance safety, it also increases the risk of opioid withdrawal. When switching opioids, clinicians must balance the risk of excessive dosing, which may lead to sedation or respiratory depression, against the risk of underdosing, which may result in poor analgesia and precipitate symptoms of opioid withdrawal.

This patient was taking tramadol for cancer-related pain prior to opioid rotation. Transitioning from tramadol to other opioid analgesics can be particularly challenging because of the lack of consensus regarding the conversion ratio between oral morphine and oral tramadol. Although oral tramadol demonstrates >90% bioavailability with chronic use [14], reported oral morphine-to-oral tramadol conversion ratios vary widely, ranging from 1:4 to 1:10 [15]. Recognizing the variability among published equianalgesic tables, an approximate 1:10 conversion ratio was used for this patient’s opioid rotation. As illustrated in this case, use of a more conservative conversion ratio during opioid rotation may contribute to the development of withdrawal symptoms.

Clinicians must be aware of tramadol’s unique pharmacology, as sudden discontinuation may precipitate a withdrawal syndrome that differs from withdrawal from other opioid analgesics. Tramadol is a centrally acting synthetic opioid that produces analgesia through a dual mechanism: mu-opioid receptor agonism and inhibition of serotonin and norepinephrine reuptake. It undergoes hepatic metabolism primarily via CYP2D6 to O-desmethyltramadol [16], which has substantially greater mu-opioid receptor affinity and potency than the parent compound [17]. This dual pharmacologic profile underlies the distinctive features of tramadol withdrawal compared to other opioids. Sudden discontinuation can produce typical symptoms of opioid withdrawal (e.g., myalgias, nausea, diarrhea, autonomic hyperactivity), as well as symptoms resembling serotonin-norepinephrine reuptake inhibitor (SNRI) discontinuation (e.g., anxiety, depression, sensory disturbances, and, in some cases, hallucinations). While typical opioid withdrawal symptoms may resolve relatively quickly with opioid replacement therapy, features resembling SNRI discontinuation, such as mood, neuropsychiatric, and sensory disturbances, may persist for weeks beyond the acute withdrawal phase [18].

Management of tramadol withdrawal is primarily supportive, utilizing antiemetics, antidiarrheals, and anxiolytics as needed. For patients experiencing prolonged monoaminergic withdrawal symptoms, there is no established evidence supporting the use of SNRIs; however, venlafaxine has been explored in limited studies for its potential role in relapse prevention among individuals with substance use disorders [19]. Tramadol withdrawal may be associated with significant noradrenergic hyperactivity, and off-label use of alpha-2-adrenergic agonists such as clonidine can attenuate sympathetic outflow, although hypotension may limit their use [20]. In patients experiencing tramadol withdrawal who require ongoing opioid therapy (e.g., for cancer-related pain), opioid dose escalation or rotation can alleviate withdrawal symptoms while maintaining adequate analgesia.

## Conclusions

Patients receiving chronic daily opioid therapy may develop tolerance, leading to diminished analgesic efficacy, for which opioid rotation is commonly employed to enhance pain control. Equianalgesic tables provide guidance for switching between different opioid analgesics and routes of administration. Ensuring safe and effective opioid transitions requires clinician familiarity with opioid pharmacology and the principles of equianalgesia and incomplete cross-tolerance. Rotation from tramadol to other opioids presents unique challenges due to variability in published conversion ratios and tramadol’s dual mechanism of action: mu-opioid receptor agonism combined with serotonin and norepinephrine reuptake inhibition.

Tramadol withdrawal can manifest features of both opioid and monoaminergic discontinuation, resulting in a complex clinical presentation that requires an individualized treatment approach. Management is largely supportive and may include antiemetics, antidiarrheals, and anxiolytics to alleviate acute symptoms. However, patients may experience prolonged mood, neuropsychiatric, and sensory disturbances related to monoaminergic withdrawal, for which evidence supporting SNRI therapy is lacking. In patients with prominent noradrenergic hyperactivity, alpha-2 adrenergic agonists such as clonidine may help attenuate sympathetic outflow, although hypotension may limit their use. When tramadol withdrawal occurs in patients who continue to require opioid therapy (e.g., for cancer-related pain), opioid dose escalation or rotation remains the cornerstone of management, ensuring adequate analgesia while alleviating withdrawal symptoms.

Effective management of chronic opioid therapy requires proficiency in opioid pharmacology and conversion strategies to mitigate risk and optimize patient outcomes. Palliative care specialists possess unique expertise in complex opioid management, making them valuable partners in the care of patients with serious illnesses such as cancer. Early referral to a palliative care team facilitates interdisciplinary collaboration, enhances safety, and supports high-quality, patient-centered care.
